# Differential Therapeutic Efficacy of Autologous Platelet‐Rich Plasma at Varying Concentrations in the Treatment of Deep Second‐Degree Burn and Its Underlying Molecular Mechanisms

**DOI:** 10.1111/jocd.70633

**Published:** 2025-12-28

**Authors:** Yu‐Fei Zhao, Yi Pan, Hong‐Qiang Liu, Fu‐Ping Zhu, Qiang Wu

**Affiliations:** ^1^ The Ninth People's Hospital of Chongqing Chongqing China

**Keywords:** deep second‐degree burn, M2 macrophage, platelet‐rich plasma, PRP at varying concentrations, transforming growth factor‐beta

## Abstract

**Background:**

Platelet‐rich plasma (PRP) shows promise in burn management, yet optimal platelet concentrations, timing, and standardized protocols remain unclear, leading to inconsistent clinical outcomes.

**Aims:**

To compare the therapeutic efficacy of PRP at different platelet concentrations in deep second‐degree burns and to explore associated molecular mechanisms.

**Patients/Methods:**

Twenty‐eight patients with small‐area deep second‐degree burns were randomly assigned to four groups: Group A (PRP 600–1000 × 10^9^/L), Group B (normal saline control), Group C (PRP 1000–1400 × 10^9^/L), and Group D (PRP 1400–1800 × 10^9^/L). Outcomes included wound healing time, wound coverage rates at 2 and 3 weeks, bacterial culture positivity, and 3‐month scar scores. Full‐thickness biopsies were collected on days 5, 9, and 16 for histopathology, immunohistochemistry, and growth factor quantification.

**Results:**

All PRP groups showed faster healing and lower infection rates compared with controls. Group C achieved the shortest healing time (*p* < 0.05). Growth factor concentrations rose with increasing platelet levels, but M2 macrophage polarization was highest in Group C. Elevated transforming growth factor‐β (TGF‐β) in Group D appeared to trigger negative feedback, limiting M2 accumulation and diminishing added benefit. Scar scores did not differ significantly among PRP groups.

**Conclusions:**

PRP accelerates wound healing and reduces infection risk in deep second‐degree burns. A platelet concentration of 1000–1400 × 10^9^/L provides the best therapeutic balance. Excessively high concentrations may impair repair through TGF‐β–mediated inhibitory effects. Future research should explore controlled‐release strategies to optimize PRP‐derived growth factor activity.

## 1 Introduction

Burn injuries rank as the fourth most common type of trauma worldwide, following road traffic accidents, falls, and interpersonal violence [1]. Beyond compromising the integrity of the skin's barrier function, burns can precipitate a systemic inflammatory response and metabolic dysregulation, which in severe cases may culminate in multiple organ failure [2]. At the initial stage of a burn injury, the body's physiological response closely resembles that of a typical inflammatory reaction. However, during the course of clinical management, this inflammatory response can be repeatedly reactivated, leading to sustained and dysregulated inflammatory cascades. Such uncontrolled inflammation may ultimately result in multiple organ dysfunction and, in severe cases, mortality [3]. In burn care, strategies have evolved from traditional wound coverage alone to a comprehensive integration of inflammation modulation, tissue function restoration, and regenerative medicine. Contemporary interventions address pain management, inflammation suppression, wound repair, and scar prevention in a coordinated manner [4]. Novel therapeutic approaches—including biomimetic adjuncts, gene‐editing technologies, and stem‐cell‐mediated signaling pathway modulation—have been developed and even introduced into clinical practice. These strategies uniformly emphasize the pivotal roles of various cytokines in promoting burn wound healing and regulating inflammation [5].

Platelet‐rich plasma (PRP) is a plasma fraction with a high concentration of platelets derived from autologous venous blood. Upon activation, PRP releases a large array of growth factors that facilitate wound healing [6]. In recent years, the application of PRP in burn management has shown remarkable potential. Through multiple mechanisms—including the release of growth factors, modulation of the inflammatory response, and promotion of tissue regeneration—PRP significantly accelerates wound healing. Numerous studies have reported its substantial efficacy in enhancing wound repair, controlling infection, and reducing late‐stage scar formation [6, 7, 8]. Currently, the research and clinical application of platelet‐rich plasma (PRP) in the treatment of burn wounds face significant challenges. Foremost among these is the general lack of reproducibility in published data, which limits the scientific validation and clinical translational value of PRP therapies. More critically, the absence of standardized protocols for PRP preparation and application further hinders its reliable use in clinical practice [9, 10]. A substantial body of research has reported inconsistent outcomes regarding the efficacy of PRP in the treatment of burn wounds. Moreover, considerable variability exists among different medical centers in terms of PRP preparation methods, platelet concentration selection, and wound management protocols [11]. Qiu et al. demonstrated through in vitro cell proliferation assays that fibroblast proliferation peaked when the platelet concentration reached 1.0–1.5 million/μL. However, further increases in platelet concentration were found to inhibit the efficiency of growth factor release [12]. In a clinical case report, Karina et al. employed an 8‐fold concentration of PRP to treat burn patients, initiating PRP intervention between days 3 and 10 post‐injury. Although favorable therapeutic outcomes were observed, the rationale behind their selected treatment parameters was not explicitly explained [13]. Some researchers have recommended maintaining PRP concentrations at 4‐ to 8‐fold levels in the treatment of various dermatological conditions [9]. However, significant inter‐individual variability exists in baseline platelet counts, and platelet levels in burn patients fluctuate over time [14]. The absence of standardized investigation into the optimal therapeutic concentration of PRP remains a major gap. Although platelet concentration is strongly correlated with growth factor release, many studies lack strict control or systematic evaluation of platelet levels, which likely contributes to inconsistent clinical outcomes [15].

Based on these considerations, a comparative analysis was designed to assess the effects of varying concentrations of platelet‐rich plasma (PRP) on infection control, wound healing duration, and scar prevention in patients with burn injuries. To ensure methodological rigor, baseline platelet levels of all enrolled patients were measured, and strict quality control of platelet concentrations in PRP preparations was implemented. Furthermore, the degree of wound epithelialization during the healing process was evaluated, immunohistochemical staining was performed, and concentrations of key growth factors were quantified to explore the underlying molecular mechanisms associated with PRP therapy.

## 2 Materials and Methods

### 2.1 Patient Population

Patients admitted to the Department of Burns at our Hospital between August 2023 and March 2025 with deep partial‐thickness (deep second‐degree) burns were recruited for the study. The inclusion criteria were as follows:

1. Age between 18 and 55 years, regardless of sex;
2. Burn injury caused by flame, scalding (hot liquid), or steam (electrical or chemical burns were excluded);
3. Classified as deep partial‐thickness (deep second‐degree) burns;
4. Time from injury to admission within 48 h;
5. Total body surface area (TBSA) involvement between 2% and 7%;
6. Clean wound surface with no prior treatment except for disinfection and simple dressing performed outside the hospital.

The exclusion criteria included:

1. Diagnosis of diabetes mellitus;
2. Heavily contaminated or clinically infected burn wounds;
3. Burns involving the head, face, neck, or perineal regions;
4. Presence of significant dysfunction in major organs such as the heart, liver, or kidneys;
5. Hypoproteinemia, defined as serum albumin < 30 g/L;
6. Thrombocytopenia (platelet count < 100 × 10^9^/L) or current use of antiplatelet medications;
7. Evidence of systemic infection, autoimmune disorders, or malignancy;
8. Use of glucocorticoids or immunosuppressive agents;
9. Diagnosis of osteomyelitis or anaerobic bacterial infections;
10. History of neurological or psychiatric disorders, or severe cognitive impairment precluding cooperation with treatment or study procedures.

We included only patients with deep partial‐thickness burns involving 2%–7% of the total body surface area (TBSA) in order to minimize the confounding effects of extensive burns, which may induce severe systemic inflammatory responses that could interfere with the study outcomes. Additionally, we excluded individuals with conditions potentially affecting the therapeutic efficacy of platelet‐rich plasma (PRP), such as thrombocytopenia or long‐term corticosteroid use. Prior to enrollment, all patients were fully informed about the treatment procedures and provided written informed consent. Relevant ethical documentation is provided in the attached files.

### 2.2 Randomization, Blinding, and Experimental Subgroup Allocation

Patients were allocated to treatment groups using a simple randomization procedure. Each patient was presented with four sealed envelopes labeled A, B, C, and D. The patient randomly selected one envelope, and the group assignment was determined according to the letter drawn, which corresponded to a specific treatment group. This clinical trial was conducted under a double‐blind design: both the patients and the physicians responsible for clinical assessments were blinded to group assignments, while the statisticians analyzing the data were unaware of the individual treatment allocations.

We established the following experimental groups to investigate the optimal therapeutic concentration range of PRP in patients with small‐area deep partial‐thickness burns by comparing treatment outcomes across different subgroups. The final PRP products may include injectable liquids, PRP gels, and PRP fibrin matrices. However, due to the complexity and high cost of preparing gel‐based formulations, as well as the challenges in quantifying and quality‐controlling platelet and leukocyte content within such products, we opted for subcutaneous injection of the liquid PRP preparation along the wound margins.

#### 2.2.1 Group A: Standard PRP Treatment Group

Patients received their first PRP treatment on day 7 post‐injury, with an injection volume of 1 mL of PRP per 1% of burn area. The concentration of the prepared PRP was standardized to 600–1000 × 10^9^/L (approximately 4 times the normal platelet count). The PRP was administered via subcutaneous injection along the wound margins using a 30 G needle, injecting 0.1 mL every 1–2 cm. A second PRP treatment was administered on day 14 post‐injury.

#### 2.2.2 Group B: Control Group

Patients received subcutaneous injections of normal saline in a manner that mimicked the standard treatment protocol, replacing PRP with sterile saline.

#### 2.2.3 Group C: Moderate‐Concentration PRP Group

Following the same protocol as the standard treatment group, PRP was prepared at a concentration of 1000–1400 × 10^9^/L (approximately 6 times the normal platelet count).

#### 2.2.4 Group D: High‐Concentration PRP Group

Following the same treatment protocol as the standard group, PRP was prepared at a concentration of 1400–1800 × 10^9^/L (approximately 8 times the normal platelet count).

### 2.3 PRP Preparation and Quality Control

On the day of PRP treatment, venous blood samples were collected from all patients in the experimental subgroups to determine baseline platelet concentrations. Based on a platelet recovery rate of 80% and a standard injection volume of 1 mL PRP per 1% of burn area, the required volume of venous blood was calculated accordingly. For example, assuming a baseline platelet concentration of 200 × 10^9^/L, a burn area of 2%, and the standard PRP group (4‐fold concentration), 10 mL of whole blood (2 mL × 4% ÷ 80%) was drawn from the median cubital vein and mixed with sodium citrate as an anticoagulant. The blood sample was first centrifuged at 1400 rpm for 10 min to separate red blood cells. The platelet‐rich supernatant was then subjected to a second centrifugation at 2800 rpm for 10 min. Based on the calculated volume, the upper layer of plasma was aspirated to yield 2 mL of standard PRP (4‐fold concentration). A 0.1 mL aliquot was retained for platelet count analysis to ensure the final concentration met the required standard.

### 2.4 Intervention Methods

After enrollment, all wound care and dressing changes were performed by physicians with at least 3 years of clinical experience. During the wound exudation phase, dressing changes were conducted 2–3 times per day, and once daily during the wound cleansing phase. Across all experimental subgroups, wound cleaning during dressing changes involved the use of povidone‐iodine combined with normal saline. Serous fluid from blisters was gently drained at low levels, while blistered skin was preserved as much as possible. Necrotic tissue and other foreign matter were carefully debrided. In cases of thick eschar formation, tangential excision was performed. After drying the wound with sterile gauze, a nanosilver‐based medical antimicrobial dressing (produced by Shenzhen AGT Pharmaceutical Technology Co. Ltd.) was applied, followed by sterile gauze coverage. The entire dressing procedure was conducted strictly in accordance with surgical aseptic principles and followed the treatment protocols outlined in international burn care guidelines [4]. Once the wounds had completely healed, all patients were advised to wear compression garments or elastic bandages continuously for 3 to 6 months to prevent hypertrophic scar formation.

### 2.5 Tissue Specimen Collection and Analysis

For all enrolled patients, wound exudate and a small amount of newly formed dermal tissue from the wound margin were collected into sterile tubes prior to dressing changes on post‐injury days 5, 9, and 16. These specimens were used for pathological examination and quantification of growth factor concentrations. Additionally, on post‐injury days 5, 9, 11, 14, and 16, wound exudate samples from the central wound base were collected prior to disinfection during dressing changes and sent for bacterial culture.

#### 2.5.1 Histopathological Examination

During dressing changes on days 5, 9, and 16 after injury, a small sample of newly formed full‐thickness skin was collected 2 mm from the advancing epithelial edge in all enrolled patients and submitted to our hospital's pathologists for histopathological evaluation and immunohistochemistry. The procedure was as follows: samples were fixed in 10% formalin for 24 h, dehydrated in absolute ethanol, and embedded in paraffin. Sections 5 μm thick were prepared using a fully automated microtome. For immunohistochemical analysis, sections were first digested with trypsin, then incubated in 3% hydrogen peroxide in methanol to block endogenous peroxidase activity. Antigen retrieval was performed in citrate buffer (pH 6.0). After washing, sections were incubated sequentially with primary and secondary antibodies according to the manufacturer's instructions, followed by DAB chromogenic development. Markers included inducible nitric oxide synthase (iNOS) and CD206. Staining was examined under 400× magnification. Positive staining, identified by yellow to brown coloration, was considered indicative of target antigen expression. Five random fields per section were selected, and the number of positive cells was counted to calculate an average. Cells expressing iNOS were classified as M1 macrophages, while those expressing CD206 were considered M2 macrophages [16].

#### 2.5.2 Growth Factor Quantification in Wound Exudate

To determine the concentration of growth factors present in wound exudate, enzyme‐linked immunosorbent assay (ELISA) was performed according to the manufacturer's instructions. The levels of vascular endothelial growth factor (VEGF), transforming growth factor‐beta (TGF‐β), and platelet‐derived growth factor (PDGF) were quantified (Registration No.: Yuxie 20202400032; provided by NewScienTech Biotechnology Co. Ltd.). Growth factor levels were determined by measuring optical density (OD) and calculating the corresponding concentrations based on standard calibration curves.

### 2.6 Evaluation Methods

#### 2.6.1 Primary Outcome

##### 2.6.1.1 Wound Healing Time

Defined as the time required for complete epithelialization of the wound, meeting the clinical standard of wound closure [17]. This parameter was assessed under double‐blind conditions by two associate chief physicians from the Department of Burns at our hospital through direct visual inspection of the wound.

#### 2.6.2 Secondary Outcomes

##### 2.6.2.1 Wound Coverage Rate at Weeks 2 and 3

The wound coverage rate was recorded at 2 and 3 weeks post‐treatment, calculated as: (Healed wound area/Total wound area) × 100%.

##### 2.6.2.2 Bacterial Culture Positivity Rate

Wound base exudate samples were collected on days 5, 9, 11, 14, and 16 for bacterial culture and antibiotic sensitivity testing. The bacterial culture positivity rate for each group was calculated as: (Number of positive cultures on days 9, 11, 14, and 16)/(Total cultures collected on those days = 4 × number of patients per group). For each patient, three cultures were randomly obtained before disinfection at the time of dressing changes; a single positive result among the three was considered indicative of a positive bacterial culture.

##### 2.6.2.3 Scar Assessment

At 3 months after wound healing, scar development was evaluated using the Vancouver Scar Scale (VSS), which includes parameters such as vascularity, pigmentation, pliability, and height.

### 2.7 Statistical Analysis

The Kolmogorov–Smirnov (K‐S) test was used to assess the normality of continuous variables, and Levene's test was employed to examine the homogeneity of variances among groups. Data meeting the assumptions of normal distribution and homogeneity of variance were expressed as mean ± standard deviation (SD). One‐way analysis of variance (ANOVA) was used for comparisons among multiple groups, and pairwise comparisons between group means were performed using the LSD‐*t* test. Categorical variables were presented as frequencies and percentages. Comparisons between two groups were conducted using the chi‐square test or continuity‐corrected chi‐square test, depending on the expected frequency. All statistical tests were two‐sided, with a significance level set at *α* = 0.05. A *p*‐value < 0.05 was considered statistically significant. All analyses were performed using SPSS version 23.0 (IBM Corp., Armonk, NY, USA).

## 3 Results

### 3.1 Patient Characteristics

The present study enrolled a total of 28 patients with deep second‐degree burns, allocated as follows: 8 patients in Group A, 5 in Group B, 8 in Group C, and 7 in Group D. At baseline, there were no statistically significant differences among the groups in terms of demographic characteristics, height, weight, or burn surface area, indicating that the groups were comparable and well‐balanced for subsequent analysis (Table 1).

**TABLE 1:** Comparison of baseline data between different treatment groups.

Group	A (*n* = 8)	B (*n* = 5)	C (*n* = 8)	D (*n* = 7)
Sex (male/female)	5/3	3/2	5/3	4/3
Age (years)	36.63 ± 13.13	39.00 ± 9.75	45.25 ± 10.80	45.57 ± 7.04
Weight (kg)	60.06 ± 9.73	62.30 ± 7.40	64.19 ± 12.10	67.64 ± 8.70
Height (cm)	168.00 ± 10.37	167.00 ± 9.59	174.25 ± 10.85	171.43 ± 10.15
Burn surface (%)	4.88 ± 1.73	4.80 ± 1.92	3.75 ± 1.98	4.14 ± 1.57
Burn site (limbs/trunk)	7/1	4/1	6/2	6/1

*Note:* There were no statistically significant differences in baseline characteristics among the groups.

### 3.2 Wound Healing and Scar Formation

Following PRP treatment, groups A, C, and D showed significantly improved wound healing outcomes compared to the control group B. Specifically, the wound healing time, wound coverage rate at 2 weeks, and wound coverage rate at 3 weeks were markedly better (*p* < 0.001), and Vancouver Scar Scale scores at 3 months were significantly lower (*p* < 0.05). Among the PRP treatment groups (A, C, D), Group C exhibited the shortest healing time and the highest wound coverage rate at 2 weeks. However, no statistically significant differences in scar scores were observed among groups A, C, and D (Table 2).

**TABLE 2:** Comparative analysis of wound healing and scar assessment scores among different treatment groups.

Group	A (*n* = 8)	B (*n* = 5)	C (*n* = 8)	D (*n* = 7)	*F*	LSD
Wound healing time (day)	19.00 ± 2.73	26.20 ± 3.27	16.63 ± 2.45	20.86 ± 2.04	14.68**	A, C, D < B**; C < A, D*
Wound coverage rate at weeks 2 (100%)	76.88 ± 11.00	41.00 ± 10.84	88.13 ± 8.43	74.29 ± 6.73	27.17**	B < A, C, D**; A, D < C*
Wound coverage rate at weeks 3 (100%)	98.13 ± 3.72	80 ± 11.18	100.00 ± 0.00	96.43 ± 5.56		
Scar assessment scores at 3 months post‐injury	7.38 ± 1.41	9.80 ± 1.48	7.13 ± 1.73	7.29 ± 1.25	4.04*	A, C, D < B*

* *p* < 0.05.

** *p* < 0.001.

### 3.3 Bacterial Culture Results

The rate of positive bacterial cultures decreased significantly after PRP treatment compared to the control group, showing statistical significance (*p* < 0.05). However, no significant differences were found in bacterial culture positivity among the different PRP concentration groups (A, C, D) (Table 3).

**TABLE 3:** Comparison of bacterial culture results among different groups.

	Bacterial culture positivity rate (Day 5)	Bacterial culture positivity rate (excluding Day 5)	Control group	*X* ^2^
A (*n* = 8)	37.5% (3/8)	40.6% (13/32)		
B (*n* = 5)	40% (2/5)	70% (14/20)	A	0.037
			C	0.023
			D	0.036
C (*n* = 8)	37.5% (3/8)	37.5% (12/32)		
D (*n* = 7)	28.6% (2/7)	39.3% (11/28)		

*Note:* Culture results on Days 9, 11, 14, and 16 were analyzed as independent samples. Bacterial culture positivity rate (excluding Day 5) = Number of positive bacterial cultures (excluding Day 5)/Total number of bacterial cultures performed (excluding Day 5), that is, 4 × number of patients per group.

### 3.4 Macrophage Polarization

**FIGURE 1:**
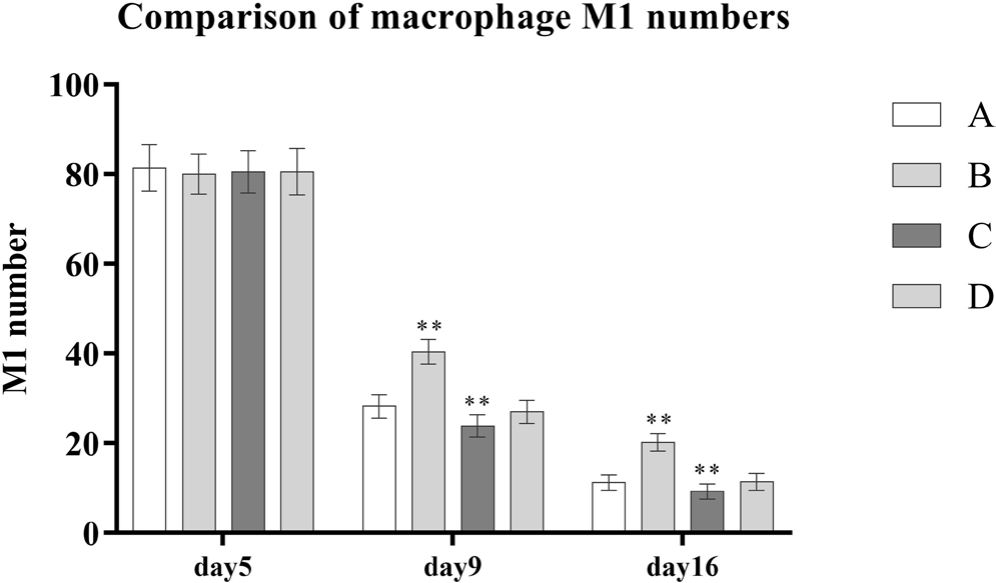
Comparison of macrophage M1 numbers.

**FIGURE 2:**
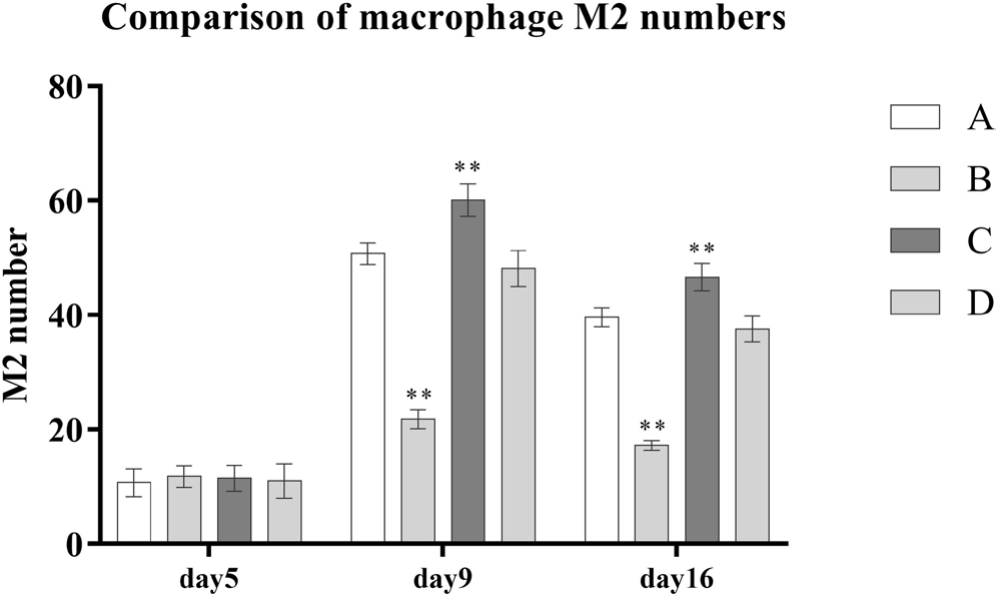
Comparison of macrophage M2 numbers.

During the wound healing process, the number of M1 macrophages in the wound tissue decreased progressively, while M2 macrophages increased. PRP treatment significantly accelerated this polarization shift. Compared to the control group B, groups A, C, and D showed significantly lower levels of M1 macrophages and higher levels of M2 macrophages (*p* < 0.001). Among the PRP‐treated groups, Group C (platelet concentration: 1000–1400 × 10^9^/L) demonstrated the most potent effect in promoting the transition from M1 to M2 macrophages, with statistically significant differences (*p* < 0.001) (Table 4 and Figures 1 and 2).

**TABLE 4:** Comparison of M1 and M2 macrophage counts across different groups.

Group	A (*n* = 8)	B (*n* = 5)	C (*n* = 8)	D (*n* = 7)	*F*	LSD
Day 5 number of M1	81.38 ± 5.21	80.00 ± 4.47	80.5 ± 4.75	80.57 ± 5.22		
Day 9 number of M1	28.25 ± 2.60	40.40 ± 2.70	23.88 ± 2.53	27.00 ± 2.58	44.16**	A, C, D < B**; C < A, D**
Day 16 number of M1	11.25 ± 1.67	20.20 ± 1.92	9.25 ± 1.67	11.43 ± 1.90	42.44**	A, C, D < B**; C < A, D**
Day 5 number of M2	10.75 ± 2.43	11.80 ± 1.92	11.50 ± 2.27	11.00 ± 3.00		
Day 9 number of M2	50.75 ± 1.91	21.80 ± 1.64	60.13 ± 2.85	48.14 ± 3.18	242.27**	B < A, C, D**; A, D < C**
Day 16 number of M2	39.63 ± 1.69	17.20 ± 0.84	46.63 ± 2.39	37.57 ± 2.23	239.18**	B < A, C, D**; A, D < C**

** *p* < 0.001.

### 3.5 Growth Factor Concentrations in Wound Exudate

**FIGURE 3:**
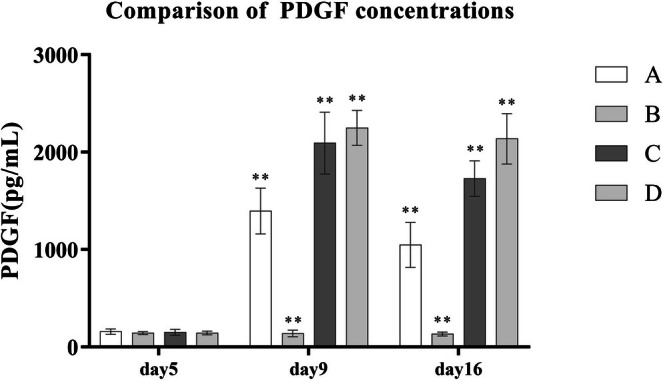
Comparison of PDGF concentrations.

**FIGURE 4:**
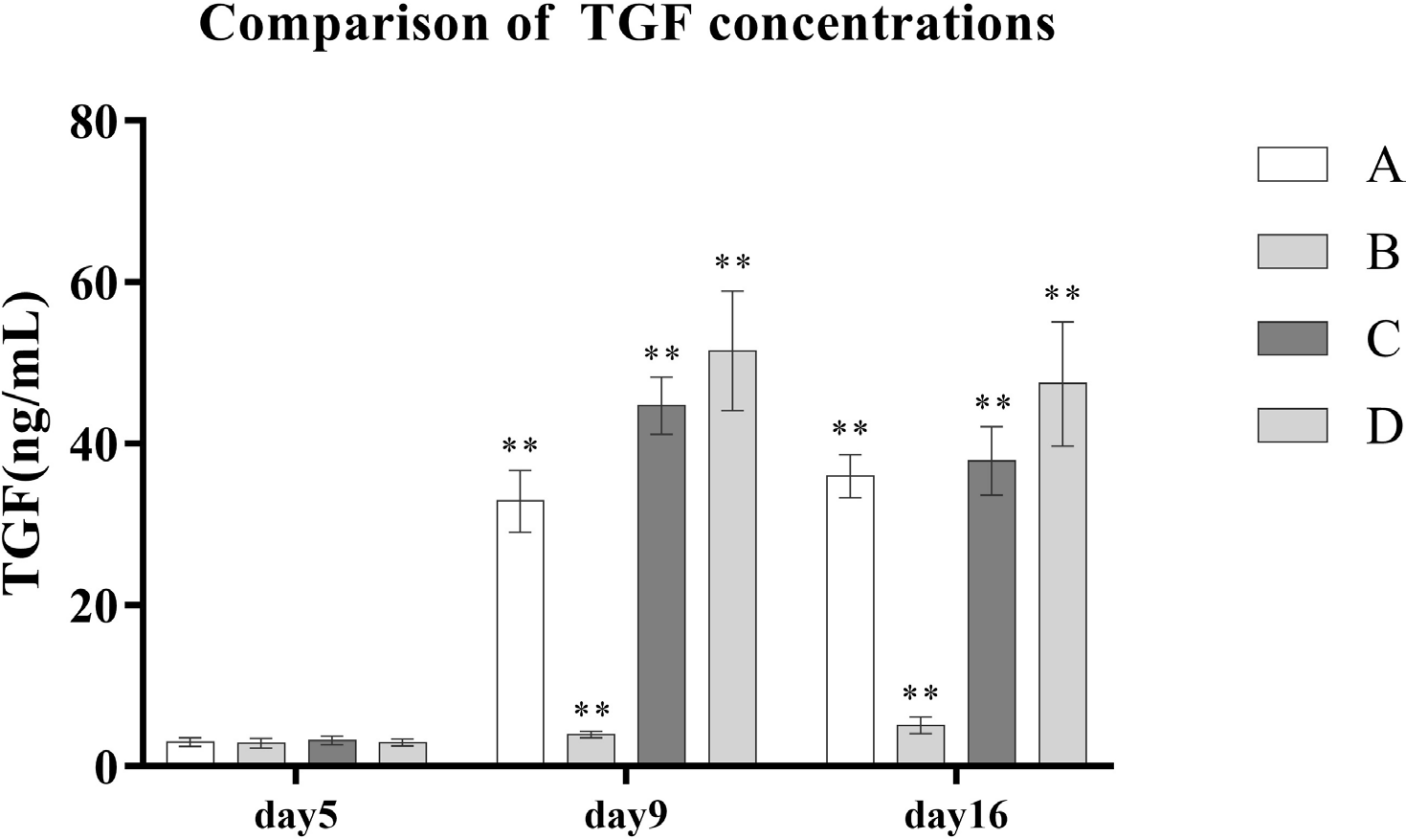
Comparison of VEGF concentrations.

**FIGURE 5:**
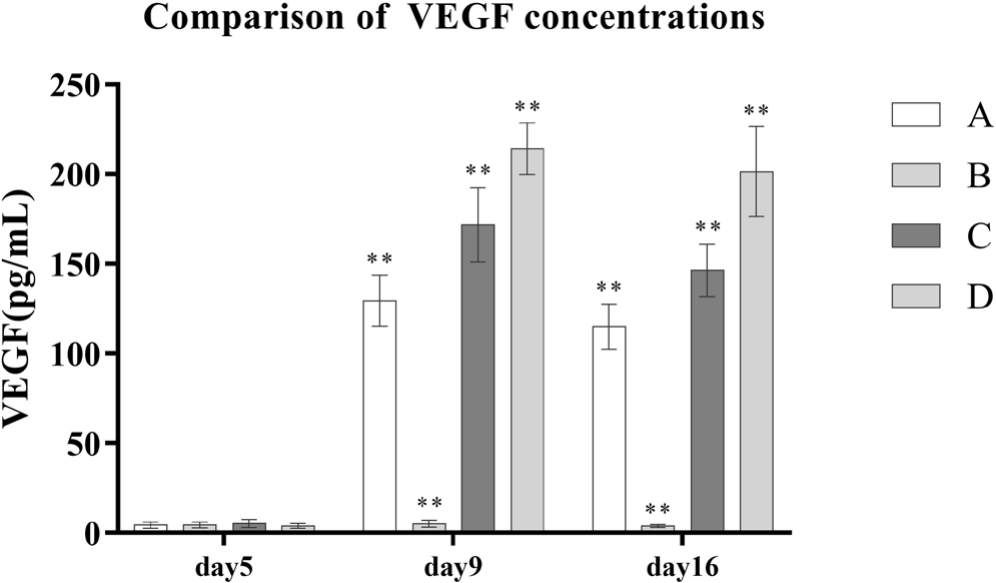
Comparison of TGF‐β concentrations.

After PRP treatment, the concentrations of key growth factors in the wound exudate—VEGF, TGF‐β, and PDGF—were significantly elevated compared to the control group B. Moreover, with increasing platelet concentrations in PRP preparations, the levels of these growth factors also rose in a dose‐dependent manner. Statistically significant differences were observed among groups A, C, and D (*p* < 0.001) (Table 5 and Figures 3, 4, 5).

**TABLE 5:** Comparison of growth factor concentrations in wound exudates across different groups.

Group	A (*n* = 8)	B (*n* = 5)	C (*n* = 8)	D (*n* = 7)	*F*	LSD
TGF‐β5 (ng/mL)	3.06 ± 0.53	2.91 ± 0.60	3.24 ± 0.51	2.99 ± 0.45		
TGF‐β9	32.88 ± 3.85	3.96 ± 0.42	44.70 ± 3.54	51.47 ± 7.40	115**	B < A < C < D**
TGF‐β16	35.97 ± 2.65	5.13 ± 1.01	37.86 ± 4.25	47.40 ± 7.68	83.64**	B < A < C < D**
PDGF5 (pg/mL)	158.00 ± 27.30	143.60 ± 14.06	151.75 ± 30.14	144.14 ± 18.92		
PDGF9	1396.13 ± 236.13	139.80 ± 32.92	2094.50 ± 317.61	2249.86 ± 179.51	98.29**	B < A < C < D**
PDGF16	1048.25 ± 230.10	134.20 ± 20.27	1729.00 ± 181.76	2138.14 ± 258.27	108.53**	B < A < C < D**
VEGF5 (pg/mL)	4.38 ± 1.69	4.40 ± 1.67	5.25 ± 2.25	3.86 ± 1.35		
VEGF9	129.50 ± 14.25	5.20 ± 1.92	171.75 ± 20.62	214.29 ± 14.37	195.94**	B < A < C < D**
VEGF16	115.00 ± 12.69	3.80 ± 0.84	146.50 ± 14.69	201.43 ± 25.09	148.14**	B < A < C < D**

** *p* < 0.001.

## 4 Discussion

Platelet‐rich plasma (PRP), a blood product with a high concentration of platelets prepared from autologous venous blood, holds substantial promise in fields such as burn and plastic surgery, dermatology, and sports medicine [11]. Alpha granules within platelets release a group of bioactive proteins known as platelet‐derived growth factors (GFs), which enhance tissue healing and regeneration. These GFs regulate and participate in a variety of physiological and biochemical processes, including tissue remodeling, angiogenesis, bone remodeling, inflammation modulation, coagulation, and cellular recruitment, growth, and differentiation [18]. PRP, as a plasma product rich in platelets, releases large amounts of GFs upon activation, thereby promoting wound healing and controlling inflammation. The concentration of these growth factors is closely correlated with the platelet count in PRP [19]. The major GFs released from platelets include platelet‐derived growth factor (PDGF), insulin‐like growth factor‐1 (IGF‐1), epidermal growth factor (EGF), transforming growth factor‐β (TGF‐β), and vascular endothelial growth factor (VEGF) [6]. PDGF promotes fibroblast migration and angiogenesis by activating the PI3K/AKT pathway [20], while EGF enhances keratinocyte migration via ERK1/2 phosphorylation and, in synergy with FGF‐2, increases cell proliferation by 1.8‐fold [21]. In a murine burn model, Bakinam M. H. Tammam [22] observed a significant increase in VEGF levels and a reduction in factors like MMP‐9 in PRP‐treated wounds, suggesting improved angiogenesis and epidermal regeneration. VEGF is one of the most potent angiogenic mediators, playing a key role in regulating vascular permeability and promoting cell migration, proliferation, and survival [23]. Numerous clinical studies have confirmed that PRP accelerates wound healing by releasing various growth factors. Consistent with these findings, our study showed that after PRP treatment in burn patients, levels of VEGF, PDGF, and other growth factors were significantly elevated in wound exudates, and histological sections demonstrated more advanced epithelialization.

However, a substantial number of studies have reported conflicting findings, which may partly explain why PRP has yet to be widely adopted in clinical practice. For example, in a prospective controlled study, Kaminski et al. [24] demonstrated that adjunctive PRP therapy significantly improved meniscal healing and function, with optimal results achieved when platelet concentration was maintained at approximately six times the baseline level. In contrast, a meta‐analysis by Migliorini et al. found that PRP combined with arthroscopic meniscal repair did not significantly enhance healing rates [25]. Notably, in the bucket‐handle tear subgroup, the PRP‐treated group exhibited a higher failure risk than the control group. The authors speculated that an excessive inflammatory response induced by PRP may have hindered tissue repair. Considerable variability in PRP preparation protocols and platelet concentrations was observed across the included studies. Similarly, a systematic review of 22 clinical trials reported that the complete healing rate of diabetic foot ulcers treated with PRP ranged from only 41% to 58%, which was substantially lower than that observed in the hyperbaric oxygen therapy group [26]. In contrast, a separate randomized controlled trial demonstrated that PRP gel significantly accelerated healing and reduced infection rates in diabetic ulcers [27]. These discrepancies likely stem from inconsistencies in PRP preparation methods, platelet concentrations, and application protocols. Moreover, suboptimal outcomes in certain studies may be attributed to excessive inflammatory activation or dysregulated expression of high concentrations of growth factors.

In the experimental results of this study, it was observed that in groups A, C, and D, as the platelet concentration in PRP increased, the concentration of growth factors in wound exudates also exhibited a sequential, inertia‐like rise. However, among these, patients in group C (with a platelet concentration of 1000–1400 × 10^9^/L, approximately six times the baseline) demonstrated the shortest wound healing time and the highest wound coverage rate at 2 weeks. This finding aligns with results from other studies, which have similarly suggested that excessive increases in platelet and growth factor concentrations may paradoxically impair tissue healing [28]. The authors hypothesize that a key underlying mechanism may involve impaired M2 macrophage polarization during tissue regeneration, as well as the detrimental effects of high concentrations of TGF‐β. Activated macrophages in wound tissues predominantly exist in two polarized states. One subtype, rich in the marker iNOS, is classified as M1 macrophages, whose primary roles include phagocytosing apoptotic cell debris, bacteria, and foreign materials, as well as secreting large amounts of pro‐inflammatory cytokines to initiate and sustain the inflammatory response at the wound site. However, in burn wounds, excessive accumulation of M1 macrophages can lead to an exaggerated inflammatory response, thereby impairing wound healing. The other subtype, characterized by high expression of the marker CD206, represents M2 macrophages, which play a pivotal role in wound repair by stimulating the differentiation, proliferation, and migration of fibroblasts, keratinocytes, and endothelial cells within the wound bed [16]. In our study, we observed that burn patients treated with PRP exhibited increased CD206 expression and decreased iNOS expression in wound tissues. This suggests that PRP promotes the phenotypic switch of macrophages from the pro‐inflammatory M1 type to the reparative M2 type, thereby facilitating wound healing. However, by comparing tissue markers and growth factor concentrations across groups with different platelet concentrations, we found that Group C exhibited the highest CD206 expression, indicating the greatest abundance of polarized M2 macrophages. Yet, the concentrations of key growth factors (VEGF, PDGF, and TGF‐β) in this group were lower than those in Group D. A substantial body of basic research has demonstrated that, during tissue repair, there exists a dynamic and interactive regulatory network between growth factor levels and macrophage populations. This synergistic network orchestrates the three key phases of healing: inflammation, proliferative repair, and tissue remodeling [29]. On one hand, high concentrations of EGF can suppress M1 markers (such as iNOS and TNF‐α) via the EGFR‐MAPK signaling pathway, while simultaneously promoting the expression of Arg1 and CD206, thereby driving the polarization of macrophages from the M1 to the M2 phenotype and fostering a reparative microenvironment. M2 macrophages, in turn, secrete critical growth factors such as TGF‐β, VEGF, and PDGF, which directly stimulate fibroblast proliferation and angiogenesis [30]. On the other hand, an excessive early presence of TGF‐β can lead to overactivation of the Smad3 signaling pathway, potentially promoting fibrotic scar formation. In bone fracture healing models, elevated local concentrations of TGF‐β have been shown to induce persistent inflammation and inhibit the accumulation of M2 macrophages [31].

TGF‐β functions as a double‐edged sword in the process of tissue repair. On the one hand, it suppresses inflammatory responses and promotes tissue regeneration by activating the Smad2/3 signaling pathway, thereby enhancing fibroblast differentiation and the chemotactic migration of mesenchymal stem cells [22]. On the other hand, excessively high concentrations of TGF‐β can lead to pathological fibrosis and excessive collagen deposition, hindering wound healing and even contributing to the formation of hypertrophic scars [32]. Our findings on scar scoring indirectly support this duality. In our study, wound healing time did not consistently correlate with the severity of scarring. Compared to the control group B, PRP treatment improved both bacterial culture negativity rates and subsequent scar hypertrophy outcomes in burn patients. However, among groups with different platelet concentrations, although patients in group C (with platelet concentrations of 1000–1400 × 10^9^/L) experienced the shortest wound healing times, there was no significant difference in scar formation between groups A, C, and D. This may be attributed to the underlying physiological mechanisms that influence scar development. In burn patients, chronic inflammation is considered one of the most critical risk factors for post‐injury hypertrophic scar formation [3]. Studies have shown that PRP can counteract burn‐induced oxidative stress in rat models by reducing the expression of pro‐inflammatory cytokines and increasing superoxide dismutase expression, thereby modulating the oxidative and inflammatory microenvironment of the wound [22]. This anti‐inflammatory effect is key to PRP's ability to reduce scar proliferation. However, excessive TGF‐β can disrupt the balance between endothelial cell proliferation and apoptosis, leading to tissue fibrosis and excessive collagen deposition, thereby offsetting some of PRP's therapeutic benefits [32]. These findings underscore that an optimal concentration range of TGF‐β is essential not only for wound healing and inflammation control but also for long‐term scar prevention. However, simply adjusting the platelet concentration in PRP to modulate the overall release of growth factors yields limited efficacy. High‐quality studies have demonstrated that adding a TGF‐β1 neutralizing antibody to PRP can enrich M2‐type macrophages, thereby promoting tissue regeneration while reducing fibrosis [32]. Some research centers have developed nanofiber scaffolds combined with PRP to prolong the half‐life of TGF‐β release. By employing a sustained‐release strategy, these systems help maintain TGF‐β within an optimal concentration range in the local tissue microenvironment—this may represent a promising direction for future research [33]. Of course, the physiological and biochemical mechanisms by which PRP promotes wound healing are highly complex. It would be overly simplistic to attribute the effects of PRP concentration on wound healing solely to TGF‐β and macrophages. In fact, different phases of burn tissue repair may require varying concentrations of TGF‐β, and the expression of distinct TGF‐β isoforms may also play critical roles. The precise regulatory mechanisms involved remain to be further elucidated through in‐depth studies.

This study has several limitations. First, the platelet concentration gradients (600–1800 × 10^9^/L) were established based on prior clinical experience with PRP therapy. Concentrations beyond this range, as well as more refined gradient intervals, may yield more robust and generalizable conclusions. Second, the study included a relatively small sample size, with only 28 participants enrolled, and was conducted at a single center, which inevitably introduces selection bias. A large‐scale, multicenter trial is warranted to validate and expand upon these findings.

## 5 Conclusion

Our study demonstrated that the use of platelet‐rich plasma (PRP) in patients with small‐area deep second‐degree burns significantly shortened wound healing time, reduced the risk of bacterial infection, and effectively improved scar formation during the later stages of recovery. Among the experimental groups with varying platelet concentrations in PRP, patients in Group C (platelet concentration of 1000–1400 × 10^9^/L) exhibited the shortest wound healing time. However, there were no significant differences among Groups A, C, and D in terms of scar formation and bacterial infection risk. At the molecular level, we observed that as the platelet concentration in PRP increased, the concentrations of growth factors in wound exudate also increased in a stepwise manner. Notably, Group C exhibited the highest degree of M2 macrophage polarization. We speculate that although elevated concentrations of growth factors such as EGF may facilitate the phenotypic transition of macrophages from M1 to M2, the early presence of high levels of TGF‐β may, through a negative feedback mechanism, inhibit M2 macrophage enrichment, thereby offsetting the beneficial effects of other growth factors. We conclude that determining an optimal concentration range for TGF‐β is critical in future PRP research. However, attempting to regulate the overall growth factor profile solely by adjusting platelet concentration may have limited benefit. Approaches such as incorporating TGF‐β sustained‐release systems or adding TGF‐β neutralizing antibodies to PRP may provide promising directions for future investigation.

## Author Contributions

Yu‐Fei Zhao performed the study and wrote the paper. Yi Pan, Fu‐Ping Zhu, and Qiang Wu collected and analyzed the data and made the tables and figures. Yi Pan and Hong‐Qiang Liu designed the study, edited the manuscript, and offered suggestions for this study. Yu‐Fei Zhao and Yi Pan contributed equally to this work.

## Funding

This work was supported by General Project of Chongqing Natural Science Foundation (Grant CSTB20233NSCQ‐MSX1062).

## Ethics Statement

The authors confirm that the ethical policies of the journal have been adhered to and the appropriate ethical review committee approval has been received. This study was reviewed and approved by the Medical Ethics Committee of Chongqing Ninth People's Hospital, with the ethical approval number Ref. No.: 2025‐KY‐(Ethics)‐015. Relevant ethical documentation is provided in the attached files.

## Conflicts of Interest

The authors declare no conflicts of interest.

## Data Availability

The data that support the findings of this study are available from the corresponding author upon reasonable request.
